# Organic Livestock Production: A Bibliometric Review

**DOI:** 10.3390/ani10040618

**Published:** 2020-04-03

**Authors:** Carmen L. Manuelian, Mauro Penasa, Luciana da Costa, Sara Burbi, Federico Righi, Massimo De Marchi

**Affiliations:** 1 Department of Agronomy, Food, Natural resources, Animals and Environment, University of Padova, Viale dell’Università 16, 35020 Legnaro (PD), Italy; carmenloreto.manuelianfuste@unipd.it (C.L.M.); mauro.penasa@unipd.it (M.P.); 2 Department of Veterinary Preventive Medicine, The Ohio State University, Columbus, OH 43210, USA; da-costa.2@osu.edu; 3 Centre for Agroecology, Water and Resilience, Coventry University, Ryton Gardens, Wolston Lane, Ryton-on-Dunsmore CV8 3LG, UK; sara.burbi@coventry.ac.uk; 4 Department of Veterinary Science, University of Parma, Via del Taglio 10, 43126 Parma, Italy; federico.righi@unipr.it

**Keywords:** bibliometrix R, cattle, organic, poultry, sheep

## Abstract

**Simple Summary:**

The organic livestock sector has been experiencing a fast growth and, lately, organic farming has become a trending topic. However, the scientific research behind the organic livestock sector is not often clear. A bibliometric review is the first approach to a topic because it helps to provide an overview of the research conducted on the topic itself. It identifies the countries involved in organic livestock production research and the scientific interaction between countries and authors, and it allows to map the keywords of the published papers. These are all key aspects to bear in mind when starting a new research area or writing a research proposal. The bibliometric analysis conducted here includes peer-reviewed documents to guarantee, from a scientific point of view, the quality of the selected studies. In this paper, we present a new technique to analyze the literature from a bibliometric point of view, and the results and conclusions extracted from the investigated topic (i.e., organic livestock production).

**Abstract:**

Due to the increasing interest in organic farming, an overview of this research area is provided through a bibliometric analysis conducted between April and May 2019. A total of 320 documents were published up until 2018 on organic livestock farming, with an annual growth rate of 9.33% and a clear increase since 2005; 268 documents have been published in 111 journals. Germany is the country with the largest number of published papers (56 documents). Authors’ top keywords (excluding keywords used for running the search) included: animal welfare (29 times), animal health (22 times), cattle (15 times), grazing (10 times), and sheep (10 times). This could indicate that more research has been done on cattle because of the importance of this species in Germany. Moreover, the prevalence of the terms ‘animal welfare’ and ‘animal health’ may indicate that the research on organic livestock production has been focused on these two areas. The bibliometric analysis indicates that: (i) countries focused the organic livestock production research on their main production, and (ii) more research in species other than cattle and sheep is needed.

## 1. Introduction

Lately organic farming has become a trending topic and research on it has increased to support the interest of consumers in organic products. An interesting approach to have an overview of the research developed on organic livestock farming is to conduct a bibliometric review which helps to identify the countries that have worked more on this topic, the most relevant authors in the field, and their connections, among others. Bibliometrics, also known as scientometrics, is based on the concept that scientific literature mirrors scientific activity because publishing papers is an important goal for the scientific community [1]. Thus, bibliometric analysis provides metrics to assess scientific interests, productivity, and impact, starting from the count of published documents and their frequency of citation [2,3]. However, it is not possible to assume a linear relationship between scientific quality and citation counts, which is the main disadvantage of that methodology [3,4]. Moreover, citations from articles to articles or from countries to countries are indicators of intellectual linkages between authors and organizations [2,3]. Co-citations analyses follow the principle that ‘the more researchers who cite the same two publications, the bigger the probability that the double citation is not a chance event’ [4], which highlights the relevance of the two references on a specific research area. Similarly, co-occurrence of keywords can be mapped and interpreted. Thus, bibliometric analysis helps understand patterns in a specific field and allows to analyze structure and dynamics. In addition, bibliometric analysis is a useful tool when planning a proposal because it highlights research hotspots and detects trends [3].

Recently, an R package (bibliometrix) was developed to easily extract the main information from a bibliometric point of view. Based on the R philosophy, detailed information of the package is available elsewhere [5]. Four main bibliographic databases (Web of Science, Scopus, Cochrane Database of Systematic Reviews, and Pubmed) can be used to extract the information to run the bibliometrix package. The Cochrane Database of Systematic Reviews focuses on systematic reviews in health care while Pubmed focuses on biomedical literature. Thus, for the topic proposed in the present paper, Web of Science and Scopus were the most adequate databases. Both databases may introduce biases in favor of natural sciences and engineering, biomedical research areas, and English-language journals [6]. The coverage of journals related to natural sciences and engineering is quite similar between both Web of Science and Scopus [6].

Therefore, the aim of this paper is to perform a bibliometric review on organic livestock farming to detect the most relevant countries, authors, cited papers, keywords, document sources, and their collaboration and co-occurrence networks.

## 2. Materials and Methods

### 2.1. Description of the Procedures Used to Select and Analyse Documents

The literature search was conducted within peer-reviewed journals as a guarantee of the quality of the selected documents. Moreover, based on preliminary search and results, we decided to keep only the information provided by Web of Science Core Collection using Web of Science v.5.32. Web of Science Core Collection fully covers 21,000 peer-reviewed, high-quality journals published worldwide in over 250 sciences, social sciences, and humanities disciplines [7]. The search in Web of Science Core Collection includes the following citations indexes: science citation index expanded (from 1985 to present), social sciences citation index (from 1985 to present), arts and humanities citations index (from 1985 to present), conference proceedings citation index-science (from 1990 to present), conference proceedings citation index-social science and humanities (from 1990 to present), and emerging sources citation index (from 2015 to present).

Keywords combinations used were: Organic + Livestock + Farming and Organic + Husbandry + Animal. We decided to minimize the keywords used to perform the search to avoid interfering with subsequent keyword analysis. The search considered the article title, abstract, authors’ keywords, and keywords Plus®. Keywords Plus® are words or phrases extracted from titles of the references cited in an article using a special algorithm developed for Clarivate Analytics [8]. The search started in April 2019 and ended on 2 May 2019 and considered a timespan for the analysis that ranged from 1985 (first year available in Web of Science Core Collection) to 2018. The ‘Organic + Livestock + Farming’ keywords combination returned 1278 documents, and the ‘Organic + Husbandry + Animal’ combination retrieved 346 documents. Thereafter, a selection based on the title and abstract was conducted to include only papers that dealt with organic livestock. Although some documents appeared in both searches, by creating a single ‘Marked list’, documents selected in one search were not allowed to be selected twice. Information regarding the documents in the marked list were exported in a BibTex file for subsequent analysis.

The analysis was carried out with the package ‘bibliometrix’ v. 2.2.0 [9] for R v. 3.6 software [10]. Briefly, the package ‘bibliometrix’ creates a database with all the information extracted (title, authors, keywords, etc.) for its analysis. It analyzes what is known as the bibliographic attributes, by creating a column for each field tag, and network structure. More updated and detailed information regarding the script is available elsewhere [11]. In addition, the last published journal impact factor (JIF; 2018) for each journal was retrieved from Journal Citation Reports provided by Clarivate Analytics and assigned to each journal in the dataset. In case the journal no longer exists, the JIF corresponds to the last year of publication of the journal. Each journal was assigned to a quartile; if a journal belonged to two or more subject categories, the category with the best quartile for the journal was considered.

### 2.2. Limitations of the Study

The criteria and the approach adopted in the present study may have some potential limitations:

- a search conducted for a bibliometric analysis does not allow to read and analyze the full paper because of the large number of documents selected;
- the search focused on peer-reviewed documents as a guarantee of the quality of the documents themselves, but this could have excluded some relevant not peer-reviewed research publications;
- only one database was used, which may result in the loss of some information. Nevertheless, the use of a single database avoids removing false duplicates when merging the selected documents from more than one database, as well as inconsistent information such as total citations of a document which may differ, albeit slightly, among different databases;
- Web of Science favors English-language journals;
- only documents with the abstract in English were included;
- fluctuations of JIF across years are greater than the ones observed for the quartile.

## 3. Results and Discussion

### 3.1. Trend of Organic Livestock Production Research

**Figure 1:**
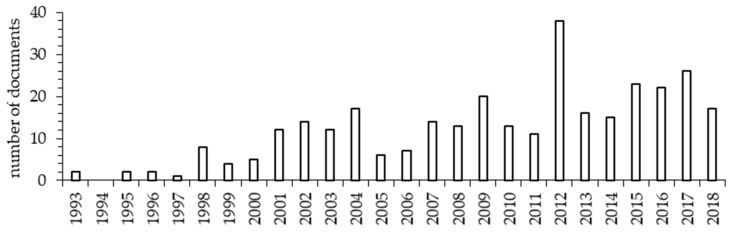
Number of documents published on organic livestock farming from 1993 to 2018 retrieved from Web of Science Core Collection using the following keyword combinations: Organic + Livestock + Farming and Organic + Husbandry + Animal.

Although the first year available in Web of Science Core Collection was 1985, the first document that accomplished the criteria of the present review was published in 1993. Therefore, the timespan of information reported in figures and tables ranged from 1993 to 2018. A total of 320 papers complied with the selection criteria. The compound annual growth rate during that period was 9.33%, with a clear increasing trend in the last years (Figure 1). It is assumed that the counts of published articles are indicative of scientific activity [2].

**Figure 2:**
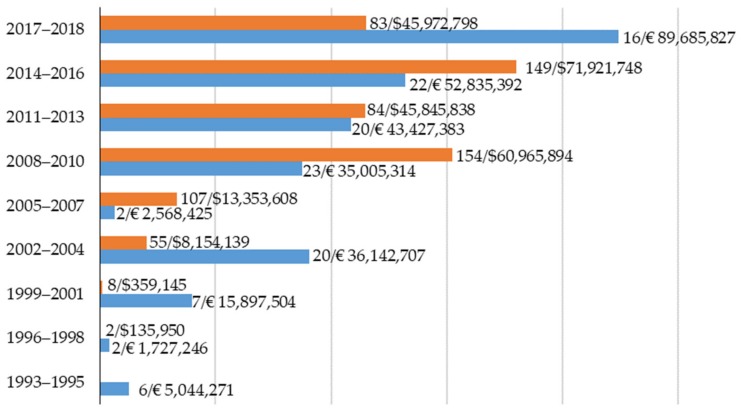
Number of projects and total funding by the European Commission (from 1993 to 2018; blue) and United States Department of Agriculture (from 1998 to 2018; orange) related to organic farming research.

Another important aspect that reveals the increasing interest of the scientific community to organic livestock production is the number of projects funded by institutions such as the European Commission (EC) and the United States Department of Agriculture (USDA). An overview of the EC funding allocation from 1993 to 2018 is depicted in Figure 2. Data were generated with a search for the keywords ‘organic’ and ‘organic farming’ on the Community Research and Development Information Service (CORDIS) and Quality of Life and Management of Living Resources (LIFE) Programme databases. While the EC maintains the Coordination of European Transnational Research in Organic Food and Farming Systems (CORE Organic) initiatives coordinated by Aarhus University (Denmark), which provides a platform for organic-only research, it also increased the number of projects and financial investment in research directly addressing issues of organic production systems. Between 1993 and 2007, the EC co-funded 37 projects on organic farming with a total investment of €57,834,611. The vast majority of the projects, however, focused on several aspects of farming, including organic practices, and only five projects were exclusively on organic livestock farming. Between 2008 and 2019, the EC co-funded 81 projects on organic farming, with a total investment of €166,738,406, and 21 projects specifically addressed organic livestock production. Gradually, the CORE Organic initiatives have become the main point of reference for organic-specific research; two livestock-specific projects were under CORE Organic in 2004, four under CORE Organic II in 2010, three under CORE Organic Plus in 2013, and six under CORE Organic CoFund in 2016. However, in the past decade there has been a growth in funding calls unrelated to CORE Organic initiatives; these calls clearly include requirements and expected impacts for the organic farming sector.

Data generated to create Figure 2 for USA projects targeted keywords ‘organic’, ‘organic livestock’, and ‘organic farming’ on the USDA database from 1998 to 2018. Briefly, the Sustainable Agriculture Research and Education (SARE) program was funded by the USDA program and included research and education grants, as well as professional development and producer grants. The Current Research Information System (CRIS) primarily focuses on National Institute of Food and Agriculture (NIFA), a federal agency within the USDA that supports broad research, education, and extension in agriculture, food, environment, and community programs. The CRIS/NIFA also provides data on researches conducted under other USDA programs such as the Organic Agriculture Research and Extension Initiative (OREI) and Organic Transitions Program (ORG). Similar to Europe, projects funded were invested in a wide range of research from soil health, food safety in organic crop, and transitioning organic livestock to sustainable agricultural production systems.

The increase of published papers and funded projects seems to follow the increasing interest for organic production by consumers and markets, including organic livestock production. Indeed, in the European Union organic livestock production increased from 1.97 × 10^6^ animals in 2000 to 41.6 × 10^6^ animals in 2015 [12]. Data from USA-certified organic cows, pigs, and sheep revealed that the number of animals increased from 56 × 10^3^ in 2000 to 492 × 10^3^ in 2011, and the number of poultry increased from 3.16 × 10^6^ to 37.0 × 10^6^ in the same period [13]. The estimated sale of organic fluid milk in the USA increased by 12% from January 2015 (210 million lbs) to January 2018 (236 million lbs; [14]).

### 3.2. Countries: Publishing Overview and Collaborations

**Figure 3:**
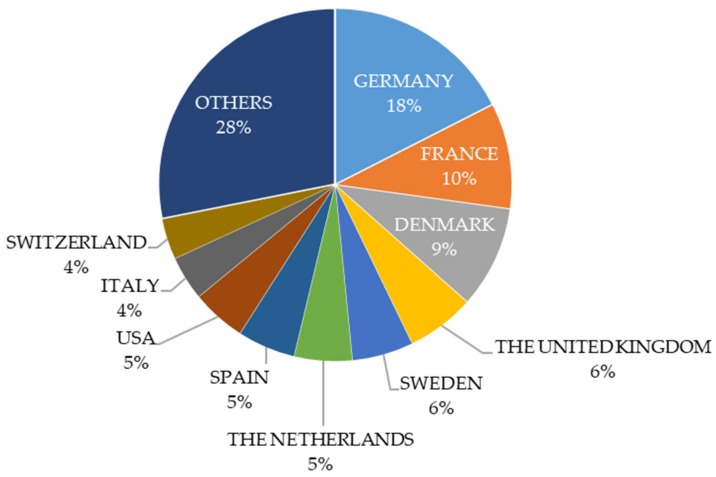
Breakdown of selected papers (*n* = 320) according to the country of the corresponding author. The group ‘OTHERS’ includes 34 countries. Documents were retrieved from Web of Science Core Collection (timespan 1993 to 2018) using the following keyword combinations: Organic + Livestock + Farming and Organic + Husbandry + Animal.

Considering the corresponding author’s affiliation, the selected documents were from 44 countries worldwide. Germany was the country with the most scientific papers published on organic livestock farming (56 documents), followed by France (31), and Denmark (30) (Figure 3). This statistic reflects the pioneer countries in organic farming. The organic farming concept started in German-speaking (Germany, Switzerland, etc.) and English-speaking countries (the United Kingdom, USA, etc.) in the early 20^th^ century due to a crisis in agriculture and agricultural science, the appearance of biologically oriented agricultural science, the Life (Germany) and Food (American) Reform movements, and the growing Western awareness of farming cultures of the Far East [15]. In addition, in German-speaking countries, the biodynamic agriculture movement started in 1924 [15]; however, biodynamic agriculture comes from an anthroposophic and spiritual view of the world [15]. At the beginning, the movement was more focused on soil fertility, plant growth, and gardening. After World War II some concepts of the Life Reform were dropped, such as vegetarianism and farming without animals, which helped to spread the organic farming concept to the mainstream of agriculture, society, and politics [15]. During the 1950s, due to the influence of British and German science-based organic farming, France embraced this movement too [15]. Therefore, countries that started the organic farming movement still account for most of the published papers.

**Figure 4:**
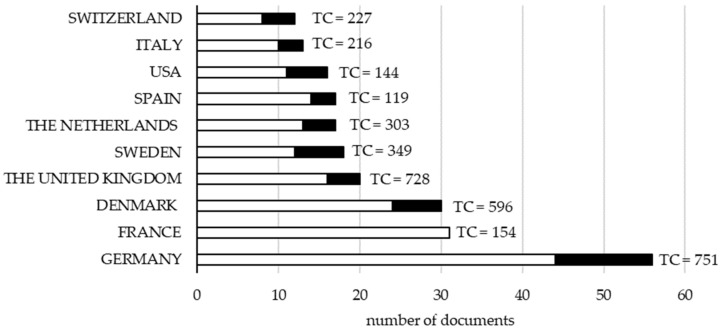
Number of documents published from 1993 to 2018 and total citations (TC) according to the country of the corresponding author (total papers = 320). Single country publications are represented by white bars and multiple country publications by black bars. Publications were retrieved from Web of Science Core Collection using the following keyword combinations: Organic + Livestock + Farming and Organic + Husbandry + Animal.

Considering the corresponding author’s country as the country of the paper, the origins of the other co-authors, based on their affiliation, were used to classify papers as single country or multiple country. Most of the documents were single country publications (255 out of 320). For example, when the corresponding author was from Germany, only 12 out of 56 documents were in collaboration with authors from other countries (Figure 4). From the 10 top producing countries, those with the greatest percentage of papers in collaboration with other countries were Sweden (31%) and Switzerland (33%), whereas when the corresponding author was from France, papers only included co-authors from France. The lack of multiple country publications for France is likely related to the fact that most papers were published in journals where the language was French, as will be discussed later.

The most cited countries, within the 320 selected documents, were Germany (751 citations), the United Kingdom (728 citations), and Denmark (596 citations; Figure 4). These results are in line with Figure 3, except for France. Indeed, the fact that the most cited documents were from Germany and the United Kingdom agrees with the long organic farming tradition of these two countries and, probably, is also related to the language in which the documents were written. Documents were mainly in English (267 out of 320), but other languages were found even if the abstract was always available in English. In particular, other languages were German (26 documents), French (20 documents), Dutch, Polish, Spanish (2 documents each), and Portuguese (1 document). The number of documents in French was close to the total number of documents from France (31 documents; Figure 4) and the number of papers published in French journals (18 papers), which supports the hypothesis that the language of the documents influences their chance to be cited. The prevalence of documents in English was expected because the search was conducted among peer-reviewed journals, which are more frequently published in English.

The total number of citations in the United Kingdom was close to that of Germany, even if Germany produced many more documents (Figure 4); this further reinforces that language has an impact on the likelihood that a document is cited. Another interesting result was that at an even number of published papers, the Netherlands had 2.5-fold more citations than Spain (Figure 4). Considering the total number of papers of the 10 top producing countries, the highest average number of citations per article was observed in the United Kingdom (36.40) and the lowest in France (4.97), which again confirms the hypothesis that non-English documents are less cited.

**Figure 5:**
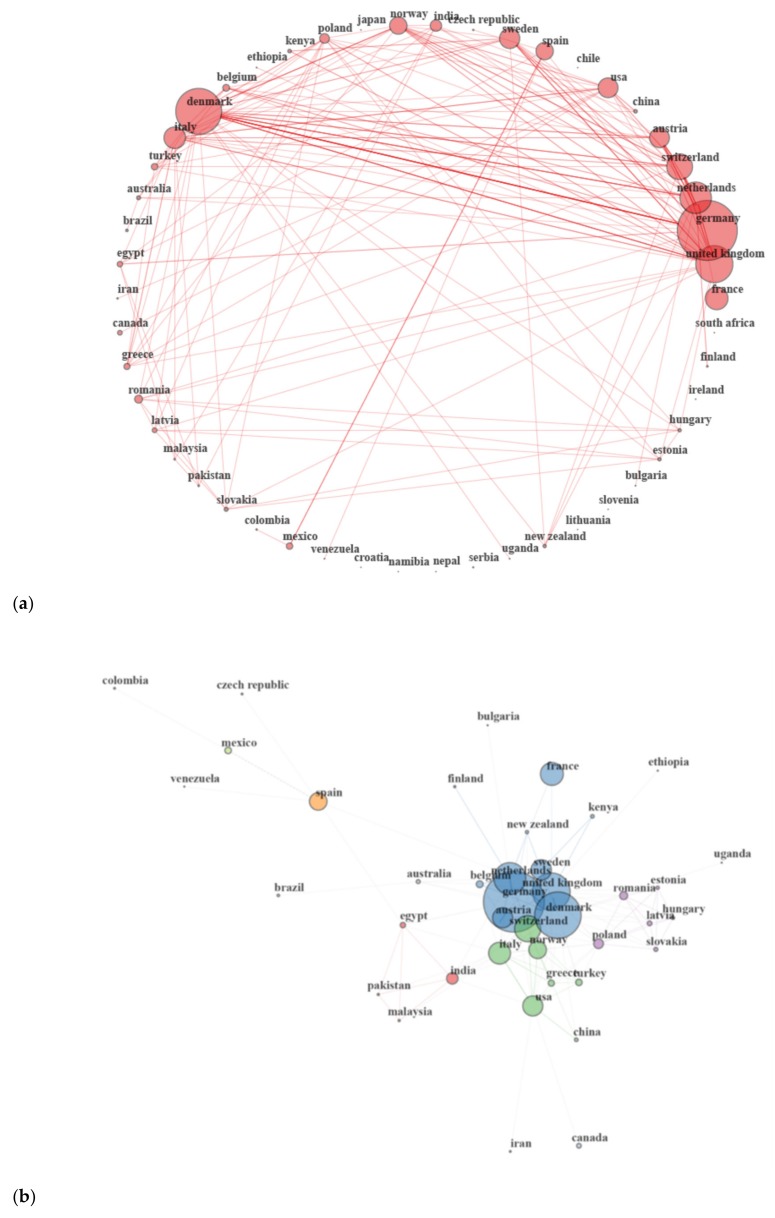
Country collaboration among countries of the database (**a**) without clusters using circle visualization and (**b**) with clusters and removing isolate vertex using Fruchterman–Reingold visualization. Each node size is proportional to the number of direct connections. The bigger the node, the more connections it has. Lines (link) between nodes represent direct connections and thickness is proportional to the number of studies involved in each direct connection. In (**b**), node colors identify clusters using the Walktrap algorithm and dash lines connect nodes of different clusters. The closer two countries are located on the map, the stronger the relation between them.

Collaborations among countries for the selected papers are depicted in Figure 5. To define collaborations, the country of each author of the paper was extracted from the affiliation [9]. Countries that presented more collaborations (biggest nodes) were Germany, Denmark, and the United Kingdom (Figure 5); this was expected because those countries published more multiple country documents (Figure 4) compared with other countries. Figure 5 reveals that French researchers were involved in publications from other countries, even if they were not the corresponding authors in any of those documents (this is also clear from Figure 4, where France does not present multiple country publications). Thus, based on the results observed for France, language used in the document impacts the possibility of collaboration among countries.

Some of the collaborations among countries can be explained based on the language or geographical proximity; for example, there was a strong collaboration between Mexico and Spain, and between Canada and the USA (Figure 5a). Strong collaboration was also observed between Germany and Denmark (Figure 5a). When using a Fruchterman–Reingold visualization (proximity of the nodes indicates stronger relation), Spain, Mexico, Colombia, and Venezuela cluster close or together (Figure 5b) which supports that language (in all these countries the official language is Spanish) and historical relationships influence the collaboration among countries. Moreover, even if Mexico and Spain are in different clusters based on the algorithm used, we can consider that all four countries are part of the same cluster because Mexico depends on the Spain node. Considering proximity of the nodes and the clusters, Poland, Slovakia, Romania, and Hungary, which are geographically close, are also in the same cluster and close in the graph, as well as Latvia and Estonia, and India and Pakistan (Figure 5b).

### 3.3. Authoring: Most Productive Authors and Collaborations

**Figure 6:**
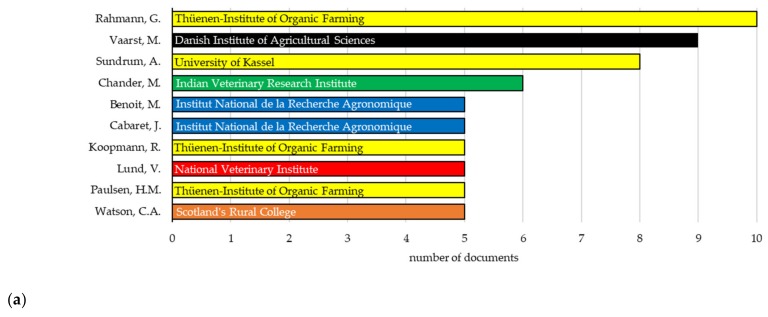

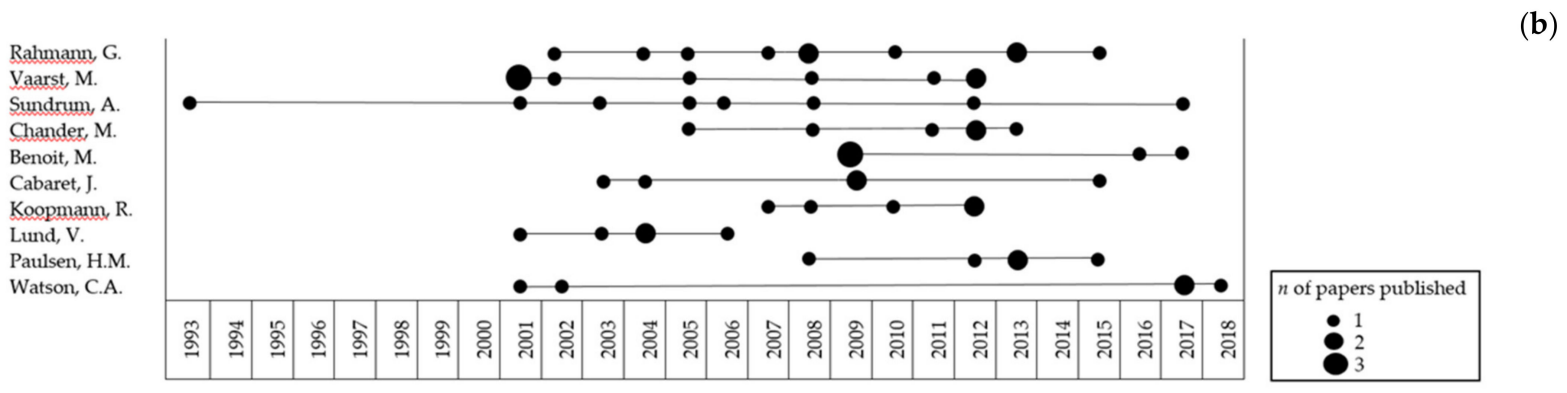
(**a**) Number of documents published by the 10 most productive authors from 1993 to 2018 on organic livestock farming. Current affiliation of the authors and country (orange, the United Kingdom; yellow, Germany; red, Norway; blue, France; green, India; and black, Denmark) are provided; (**b**) Papers published by the 10 most productive authors in a timeline scale. Publications were retrieved from Web of Science Core Collection using the following keyword combinations: Organic + Livestock + Farming and Organic + Husbandry + Animal.

Most of the selected papers (275 out of 320) were contributed by several authors, and only 14% of them were single-authored documents. A total of 1000 authors were identified, from which 38 were authors of single-authored documents and 962 of multi-authored documents. Considering only the authors of multi-authored documents and documents with more than one author [16], the collaboration index was 3.5, which implies that the average research team falls between three and four in the field of study. Within the 320 selected documents, the 10 most productive authors, in terms of number of published papers and based on their current affiliation, were from Germany (4 authors), France (2 authors), and Denmark, India, Norway, and the United Kingdom (1 author from each country; Figure 6a). In addition, the number of documents per author ranged from 5 to 10 considering those 10 authors.

In terms of number of published documents per author, G. Rahmann [17] was the main author who has published papers on the topic covered from 2002 to 2015. In terms of longevity, A. Sundrum [18] covered the widest timespan (1993 to 2017), publishing papers related to organic livestock production. Thünen Institute of Organic Farming [19] in Germany (G. Rahmann, R. Koopmann, and H.M. Paulsen) conducts interdisciplinary research in organic farming, mainly in organic livestock farming and crop production. Organic farming and, in particular, organic livestock research is also covered by M. Vaarst [20], M. Chander [21], M. Benoit [22], and J. Cabaret [23]. However, J. Cabaret has dealt with parasitology research [23].

Moreover, Figure 6b highlights that some authors have not been involved in organic livestock production research so far. For example, V. Lund was a specialist in animal welfare and animal ethics whose thesis was related to organic animal husbandry and this explains the five articles included [24]. In addition, Figure 6 reveals interdisciplinary research groups when publishing in organic livestock. For example, C.A. Watson’s research area was related to soil science [25]. However, the documents co-authored by C.A. Watson in our database (*n* = 5) were organic farming reviews also covering livestock or research papers with crop-livestock farms. This was somehow expected because, as indicated above, the ‘organic farming’ movement started in soil and plant science, and a more holistic approach is used than in conventional farming [15].

### 3.4. Most Cited Documents, Used References, and Co-citations

**Figure 7:**
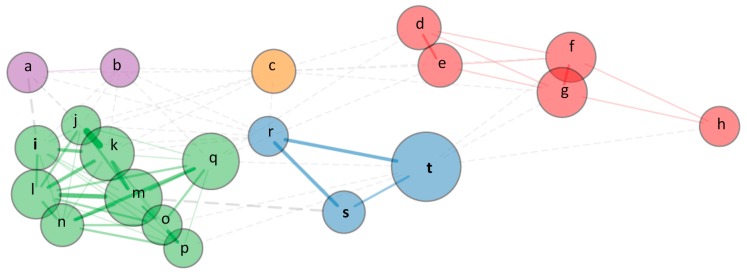

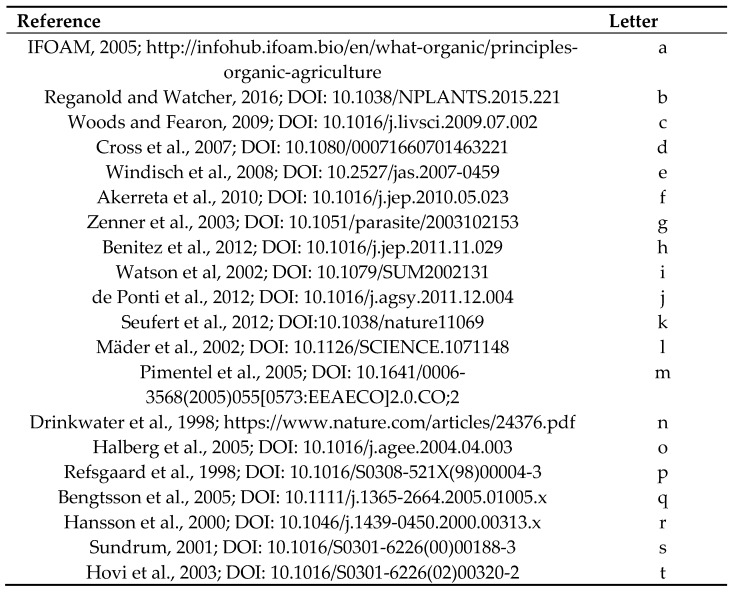
References co-citation network of the top 20 references using Fruchterman–Reingold visualization. Each node size is proportional to the number of direct connections. The bigger the node, the more connections it has. Lines (link) between nodes represent direct connections and thickness is proportional to the number of studies involved in each direct connection. Node colors identify clusters of references using the Walktrap algorithm. Dash lines link nodes of different communities. The closer two references are located on the map, the stronger the relation between them.

Among the 320 selected documents, the top 10 with the greatest number of citations up until May 2019 are listed in Table 1. Considering only the top five, four documents were tagged as reviews, which supports that the most important citing reason of a reference is reviewing prior work on the topic [1]. Nevertheless, the classification of a document as an article or a review is sometimes questionable. Although we used the metadata linked to the document regarding the type of document, we realized that some documents tagged as ‘articles’ were in fact ‘reviews’. For example, the paper of M. Hovi as first author (Table 1) was a review that described the state and the future challenges of organic livestock in Europe. The number of citations of a paper depends on the year of publication, among other things, and most recent papers have had less of a chance to be cited, which partially explains why the year of publication of the most cited papers ranged from 1998 to 2011 (Table 1a). The number of citations usually indicates the impact (influence) of the article [2]. However, as already mentioned, citations are not a synonym of quality, and they depend also on the type of research and long-term significance for the scientific community [4]. Methodological articles and reviews are cited more often than other papers; in addition, papers considered of bad quality are cited more than papers considered mediocre due to ‘negational citation’ [4]. Therefore, more than the number of times an article is cited, it is interesting to consider the number of articles (in our database, 320) that used the same reference (Table 1b) or co-citation analysis (Figure 7).

The 10 most used references within our selected database were documents from 2000 to 2006 (Table 1b). In particular, the most used reference cited by 17 out of the 320 documents was published by M. Hovi as first author (Table 1), followed by the paper of T.W. Bennedsgaard as first author (Table 1b). Only one of the most used references was tagged as a review article (Table 1b). However, as indicated above, some documents classified as ‘articles’ are in fact ‘reviews’. Along with the paper of M. Hovi as first author, those from J.E. Hermansen, E. Von Borell, and H.F. Alrøe as first authors, were also reviews (Table 1b). Therefore, half of the references in Table 1b are reviews, and two of them are within the top five of the list.

Co-citations of the references within a specific area of research allow to identify the most relevant references. They indicate the frequency with which two documents are cited together by other documents [26]. Those patterns can vary over time as a result of changes in interests and intellectual patterns of a field [26]. Co-citation analysis of the 20 most co-cited references revealed five clusters (Figure 7). The red cluster includes two studies on ethnoveterinary (letters f and h), and papers that dealt with the use of plants in livestock (letters d and e) or in vitro studies with essential oils (letter g). The green cluster includes studies more related to agriculture (soil, environmental studies, and crops; letters i to k). The blue cluster identifies papers more focused on livestock (letter s and t) or quality of animal-derived products (letter r). The biggest nucleus (letter t in Figure 7), which means that it is the most co-cited, refers to the review published by M. Hovi as first author. This document was also the most cited in our dataset (Table 1b), which confirms that two documents frequently co-cited are also frequently cited individually [26]. Moreover, this review (letter t in Figure 7) was written in collaboration with A. Sundrum, who is one of the most productive authors in our dataset (Figure 6). Nucleus ‘s’ (Figure 7) refers also to a review paper published by A. Sundrum about organic livestock farming (Table 1). Furthermore, A. Sundrum is the author who has published on that topic for a longer time, and he is currently affiliated with a university in Germany (Figure 6).

### 3.5. Documents Sources

**Figure 8:**
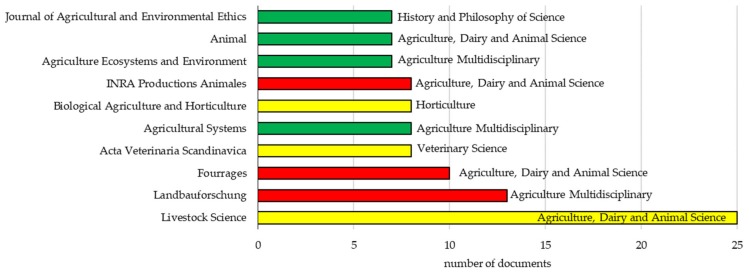
The 10 journals with more papers published from 1993 to 2018 on organic livestock farming. The quartile of the journal (quartile 1, green; quartile 2, yellow; quartile 3, orange; and quartile 4, red) is based on the 2018 Journal Impact Factor (JIF) published by Clarivate Analytics. When the journal was included in several categories, the one with the best quartile was considered. Categories considered to establish the quartile are also included in the graph. Publications were retrieved from Web of Science Core Collection using the following keyword combinations: Organic + Livestock + Farming and Organic + Husbandry + Animal.

The 320 documents were published in 111 journals (268 articles), 25 proceeding books (51 articles), and a bulletin (1 article). The sources identified in our study mirror the characteristics of the database used to conduct the search [6]. Based on 2018 JIF, 38 of those journals were from the 1^st^ quartile of their category, 26 from the 2^nd^ quartile, 14 from the 3^rd^ quartile, and 28 from the 4^th^ quartile. In addition, 63.8% of the papers were published in 1^st^ and 2^nd^ quartile journals (93 and 78 documents, respectively), and 27% were published in 4^th^ quartile journals (72 documents). Only 5 out of 268 papers were published in journals without a JIF in 2018. The 10 journals with the most published papers are presented in Figure 8 and accounted for 101 out of 320 documents. Moreover, the journals in the top three positions are classified nowadays in the 2^nd^ (Livestock Science) and 4^th^ (Fourrages and Landbauforschung) quartiles of their category, and account for 40 papers.

From the top 10 journals, Fourrages and INRA Productions Animales are published in French, and included 18 out of the 320 documents, which could partially explain the fact that Fourrages and INRA Productions Animales are in the 4^th^ quartile of their category, and the lack of multiple country publications when France is the country of the corresponding author (Figure 4). Landbauforschung and Acta Veterinaria Scandinavica are classified as multi-language journals (use of more than one language) by Web of Science. The use of German and English in Landbauforschung was confirmed by visiting the journal website; however, in the website of Acta Veterinaria Scandinavica, papers were only in English. The low rank of those journals in their respective category supports that publishing in languages different to English impairs the impact factor because there is lower visibility of the papers.

Nowadays, JIF is considered a quality parameter of published research with regard to assessment and prioritizing the allocation of funds [4]. However, because JIF is calculated on the basis of the previous two years, it favors journals whose papers are cited intensively for a very short period after their publication, but not in the long term [4]. Moreover, JIF is influenced by the type of articles that the journal publishes (original research papers, reviews, etc.), the number of articles published in that journal during the previous two years, and the journal accessibility (which includes language of the articles) [4]. However, the use of quartile distribution allows to compare journals across categories. Most of the proceeding papers (42%) were published in the book of abstracts of the 2^nd^ Organic Animal Husbandry conference that took place in Hamburg in 2012.

### 3.6. Most Important Authors’ Keywords and Co-occurrence

A total of 842 authors’ keywords were extracted from the documents. Without editing synonyms, the authors’ top 10 keywords were: ‘organic farming’ (89 times), ‘animal welfare’ (29 times), ‘organic’ (25 times), ‘animal health’ (22 times), ‘organic agriculture’ (17 times), ‘cattle’ (15 times), ‘livestock’ (12 times), ‘organic livestock production’ (12 times), ‘grazing’ (10 times), and ‘organic production’ (10 times). ‘Sheep’ was also used 10 times. It is important to bear in mind that the words used to conduct the search (in our case, Organic + Livestock + Farming and Organic + Husbandry + Animal) are overrepresented. For example, we expect an overrepresentation of the terms ‘organic farming’, ‘organic’, ‘organic agriculture’, ‘livestock’, ‘organic livestock production’, and ‘organic production’. Therefore, the top 10 keywords when excluding those overrepresented terms were: ‘animal welfare’ (29 times), ‘animal health’ (22 times), ‘cattle’ (15 times), ‘grazing’ (10 times), ‘sheep’ (10 times), ‘grassland’ (9 times), ‘health’ (9 times), ‘dairy’ (8 times), ‘sustainability’ (8 times), and ‘forage system’ (7 times). Poultry was also used 7 times. The presence of ‘cattle’ among the authors’ top 10 keywords is in line with Germany being the country that has published the greatest number of articles on organic livestock farming and has the greatest number of organic cattle. The presence of ‘poultry’ in the top 10 keywords (after removing the words used to conduct the search) was not surprising considering it is by far the most important species reared organically in Europe with a share of 76% in 2015. Based on information from the USDA and accredited state and private organic certifiers, the growth of certified organic operations/number of birds from 2000 (6592/3,159,050) to 2011 (12,880/37,028,242) demonstrates not only the importance of the poultry industry (layer hens, broilers, and turkeys) to the economy of the USA, but also the increased interest in organic production for this commodity [13]. Although Denmark is the second most prolific country in terms of scientific papers and the leader in organic pig production, ‘pig’ or any related word was not represented in the authors’ top 10 keywords.

**Figure 9:**
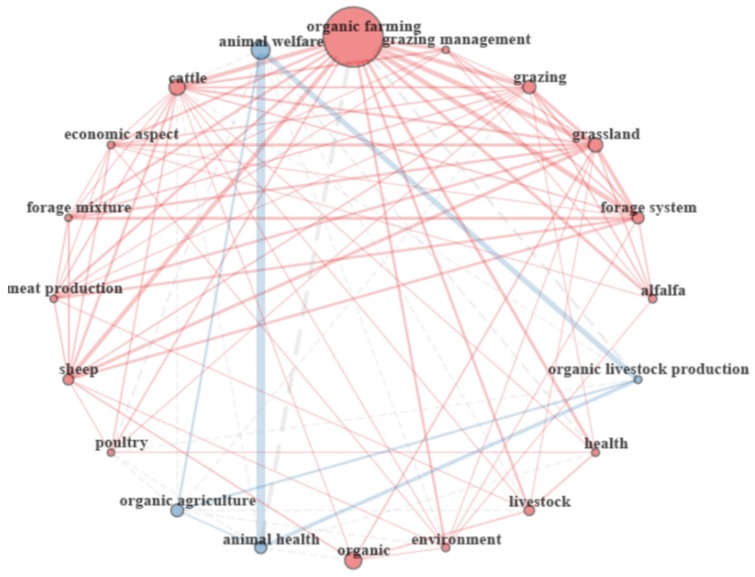
Network plot of the top 20 authors’ keywords co-occurrence using circle visualization. Each node size is proportional to the number of direct connections. The bigger the node, the more connections it has. Lines (link) between nodes represent direct connections and thickness is proportional to the number of studies involved in each direct connection. Node colors identify clusters of words using the Walktrap algorithm. Dash lines link nodes of different communities.

As indicated for the co-citation analysis, co-recurrent keywords are interpreted in a similar way. The more times that the same two words are listed in the keywords section of an article, the more we can rely that they are not there by chance. The analysis of the keywords co-occurrence gives an overview of the research performed. The analysis of the 20 more co-recurrent keywords revealed the presence of two clusters (red and blue; Figure 9); the blue one included the terms ‘animal welfare’, ‘organic agriculture’, ‘animal health’, and ‘organic livestock production’. The most co-occurring keywords among papers were ‘organic farming’ and ‘animal welfare’, being both the principal keywords in their cluster. Most papers combined the keyword ‘animal welfare’ with ‘animal health’ and ‘organic livestock production’; and ‘organic farming’ with ‘cattle’, ‘grazing’, ‘grassland’, ‘forage system’, and ‘sheep’. However, intra-cluster connection was also observed; indeed, the term ‘organic farming’ was strongly connected with ‘animal health’. This analysis highlights the relation of welfare with health, organic production with pasture, and organic production with animal health and welfare. As previously stated, it is likely that the words ‘organic farming’, ‘organic livestock production’, ‘livestock’, ‘organic’, and ‘organic agriculture’ were overrepresented because of the word combination used for conducting the search.

Only three species appeared in the top 20 co-occurring keywords: ‘cattle’, ‘sheep’, and ‘poultry’, with ‘cattle’ as the most important. The presence of links among those species, although scarce, suggests multi-species studies. Studies seemed more focused on feeding, mainly forage and pasture, than on other issues. Regarding animal products, only ‘meat’ was present in the co-occurring keywords analysis. However, ‘cattle’ was one of the terms with the biggest nucleus, which could reflect the relevance of organic milk. In fact, organic milk production has almost doubled since 2007, from 2.7 to 4.7 million metric tons [27]. This could be related to the fact that animal health and welfare, and not product quality, were the main areas of research detected in Figure 9. However, it has to be taken into consideration that some important keywords could be in the title of the document and not in the authors’ keyword list, and guidelines of some journals recommend to use terms in the keywords list that differ from those in the title.

## 4. Conclusions

The bibliometric approach offered an overview of the topic of our study—organic livestock production. It allowed us to manage a huge amount of references to highlight tendencies and science mapping, such as topics investigated, most prolific authors and countries, co-citations of the references, and co-occurrence keywords. Our study reveals an increasing interest in organic livestock production, which can be scientifically measured by the number of funded projects by governmental organizations and the number of published papers. Countries with a long-tradition in organic farming (German-speaking and English-speaking countries, and France) are still the predominant countries in organic livestock research. Our results also highlight the impact of the language used to write the papers on their visibility and scientific impact (e.g., citations and JIF of the journal). Collaborations among countries are still scarce, and probably triggered by geographical and historical relations and languages. It also seems that multidisciplinary research including authors from several research areas (e.g., ethnoveterinary, plants/soil science, in-vitro studies, economic studies) is conducted when investigating organic livestock production. Some limitations of the search (e.g., database used, keywords, software for the analysis, language of the documents) and metadata linked to the documents (e.g., type of document, total citations) have to be accounted for when interpreting the results. Therefore, a bibliometric review is an interesting and reliable way to have an overview of a specific topic being approached for the first time.

## Figures and Tables

**Table 1:** (**a**) The 10 most cited papers among the 320 selected documents on organic livestock farming. First author name, year of publication, digital object identifier (DOI), total citations, and type of document. Total citations were extracted on May 2019. (**b**) The 10 most used references on organic livestock farming in the 320 selected documents. First author name, year of publication, DOI, number of times the reference was cited, and type of document.

**(a) Document**	**Total Citations**	**Type of Document**
G. Haas, 2001; DOI: 10.1016/S0167-8809(00)00160-2.	273	Article
C.A. Watson, 2002; DOI: 10.1079/SUM2002131.	200	Review
G. Huyghebaert, 2011; DOI: 10.1016/J.TVJL.2010.03.003.	190	Review
E.A. Stockdale, 2011; DOI: 10.1016/S0065-2113(01)70007-7.	175	Review
A. Sundrum, 2001; DOI: 10.1016/S0301-6226(00)00188-3.	171	Review
T. Nemecek., 2011; DOI: 10.1016/J.AGSY.2010.10.002.	128	Article
J.E. Olesen, 2006; DOI: 10.1016/J.AGEE.2005.08.022.	124	Article
K. Refsgaard, 1998; DOI: 10.1016/S0308-521X(98)00004-3.	120	Article
M. Hovi, 2003; DOI: 10.1016/S0301-6226(02)00320-2.	101	Article ^1^
N.E.H. Scialabba, 2010; DOI: 10.1017/S1742170510000116.	92	Review
**(b) Document**	**Times Cited by the 320 Documents**	
M. Hovi, 2003; DOI: 10.1016/S0301-6226(02)00320-2.	17	Article ^1^
T.W. Bennedsgaard, 2003, DOI: 10.1016/S0301-6226(02)00312-3.	13	Article
A. Sundrum, 2001; DOI: 10.1016/S0301-6226(00)00188-3.	11	Review
F. Hardeng, 2001; DOI: 10.3168/JDS.S0022-0302(01)74721-2.	10	Article
I. Hansson, 2000; DOI: 10.1046/J.1439-0450.2000.00313.X.	9	Article
J.E. Hermansen, 2003; DOI: 10.1016/S0301-6226(02)00313-5.	9	Article ^1^
E. Von Borell, 2004; DOI: 10.1016/J.LIVPRODSCI.2004.07.003.	9	Article ^1^
H.F. Alrøe, 2001; DOI: 10.1023/A:1012214317970.	8	Article ^1^
A. Busato, 2000; DOI: 10.1016/S0167-5877(00)00104-5.	8	Article
W.J. Nauta, 2006; DOI: 10.1016/J.LIVSCI.2005.06.013.	8	Article

^1^ classified as an ‘article’ by the metadata linked to a document, but the paper is in fact a review.
